# Assessing the Feasibility and Utility of Patient-Specific 3D Advanced Visualization Modeling in Cerebrovascular Disease: Retrospective Analysis and Prospective Survey Pilot Study

**DOI:** 10.2196/51939

**Published:** 2025-02-21

**Authors:** Korak Sarkar, Vishal Bhimarasetty, Abdul Rahim, Colin Curtis, Kimberly Hughes

**Affiliations:** 1 Department of Academics Ochsner BioDesign Lab Ochsner Health New Orleans, LA United States; 2 Ochsner Neuroscience Institute Ochsner Health New Orleans, LA United States; 3 Physical Medicine and Rehabilitation Service Southeast Louisiana Veterans Health Care System New Orleans, LA United States; 4 University of Queensland School of Medicine Queensland Australia; 5 Ochsner Information Services Ochsner Health New Orleans, LA United States

**Keywords:** cerebrovascular disease, advanced visualization, 3D modeling, cerebrovascular, intracerebral arteriovenous malformations, artery, vein, vessel, medical extended reality, 3D printing, medical simulation, virtual reality, augmented reality, usability, survey, stroke, brain, cerebral

## Abstract

**Background:**

The prevalence, clinical burden, and health care costs (>US $100 billion) associated with cerebrovascular disease (CVD) will increase significantly as the US population grows and ages over the next 25 years. Existing 2D imaging modalities have inherent limitations in visualizing complex CVD, which may be mitigated with the use of patient-specific 3D advanced visualization (AV) technologies. There remain gaps in knowledge, however, regarding how and with what impact these technologies are being used in CVD.

**Objective:**

The aim of this study was to characterize the clinical attributes and reported utility associated with the use of 3D AV modeling in CVDs, specifically intracerebral arteriovenous malformations.

**Methods:**

This pilot study employs a combination of retrospective analysis and prospective surveys to describe the utilization and utility of patient-specific AV models at a single high-volume certified comprehensive stroke center.

**Results:**

From July 2017 to February 2023, 25 AV models were created for 4 different clinicians. The average patient age was 37.4 years; 44% (11/25) of the patients were African Americans, 52% (13/25) were on public insurance, and 56% (14/25) were associated with a neurovascular procedure. In this study, 18 clinicians with diverse experience responded to AV model surveys, with a 92.2% (166/180) completion rate. There was an average reported utility of 8.0 on a 0-10 scale, with higher scores reflecting increased utility. Compared to 2D viewing, AV models allowed staff to appreciate novel abnormal anatomy, and therefore, they would have changed their therapeutic approach in 45% (23/51) of the cases.

**Conclusions:**

AV models were used in complex CVDs associated with young, publicly insured individuals requiring resource-intensive interventions. There was strong and diverse clinician engagement with overall report of substantial utility of AV models. Staff clinicians frequently reported novel anatomical and therapeutic insights based on AV models compared to traditional 2D viewing. This study establishes the infrastructure for future larger randomized studies that can be repeated for CVDs or other disease states and incorporate assessments of other AV modalities such as 3D printing and medical extended reality.

## Introduction

### Background

Cerebrovascular disease (CVD) is a common cause of stroke, aneurysms, and thromboembolic disease, resulting in significant neurologically based disability and death [1,2]. Although CVD can be treated with medications, many disease presentations require procedural interventions. It is estimated that by 2030, the annual costs for cerebrovascular accidents alone will exceed US $140 billion [3]. Improved visualization, greater repetition, and more frequent training experiences are factors known to improve outcomes in CVD treatments requiring complex procedures [4]. Treatment of patients with difficult or otherwise hostile anatomies carries more risk when operators have less general experience navigating unusual or atypical structures [5]. The lack of anatomical and procedural familiarity can lead to lengthier procedures, longer anesthetic duration, increased radiation exposure, and poorer outcomes [5,6]. Access to personalized 3D models designed for patient-specific planning can provide higher fidelity visualization and simulation than traditional 2D viewing, thereby reducing the prevalence, associated costs, and morbidity from complex CVD procedures [7].

The rapid adoption of advanced visualization (AV) tools such as 3D rendering, 3D printing, and medical extended reality has created novel opportunities for medical visualization and simulation. Patient-specific 3D digital and physical anatomical models based on clinical radiographic data can now be rendered and fabricated for patient education, clinical training, and procedural planning [8]. This approach holds great promise as a tool in visualizing complex neurovascular anatomy and simulating complex surgical and endovascular procedures [9]. The increasing adoption of advanced modeling has prompted organizations such as the Radiological Society of North America to issue guidelines for implementing medical 3D printing as training models for highly complex clinical scenarios [10]. The Radiological Society of North America guidelines discuss the use of patient-specific 3D models for a variety of specialties, including congenital heart disease, craniomaxillofacial, breast, and musculoskeletal pathologies. There, however, remains limited guidance on the optimal use of 3D modeling in CVD.

CVD interventions are an ideal application of AV modalities due to the idiosyncratic and patient-specific 3D nature of intracerebral vascular pathology. Current evidence suggests that AV technologies such as 3D rendering, medical extended reality, and 3D printing are an appropriate platform for studying and rehearsing plans for treating complex CVD [11]. Several studies have reported the growing utilization of AV modeling, visualization, and procedural training in CVD [12]. The current state of research, however, remains limited and warrants more thorough investigation [12,13]. To address this gap, the field would benefit from the development of a scalable modeling and assessment platform, which could describe and evaluate the use of patient-specific AV anatomical modeling. Such an infrastructure could address the clear, critical, and well-identified need to characterize the use and utility of AV models in CVD.

### Objectives

This pilot study was designed and executed to describe the utilization and utility associated with the use of patient-specific anatomical models in the management of intracerebral arteriovenous malformations (iAVMs). This exploratory investigation involves the implementation of a scalable digital fabrication infrastructure to produce patient-specific anatomical AV models, characterize their use, and quantify their potential impact in the management of iAVMs. The aims of this study include describing the clinical, anatomical, and demographic features associated with the use of patient-specific AV models in the management of iAVMs. Additionally, this study aims to quantify the utility of patient-specific AV anatomical models when compared to traditional 2D viewing across different clinical experience levels. We hypothesize there is a negative correlation between experience and reported utility of AV patient-specific anatomical models in CVD. We specifically predict that when compared to standard 2D viewing, clinicians with less experience will report higher utility of AV patient-specific anatomical models than more experienced staff clinicians.

## Methods

### Digital Fabrication Process

**Figure 1:**
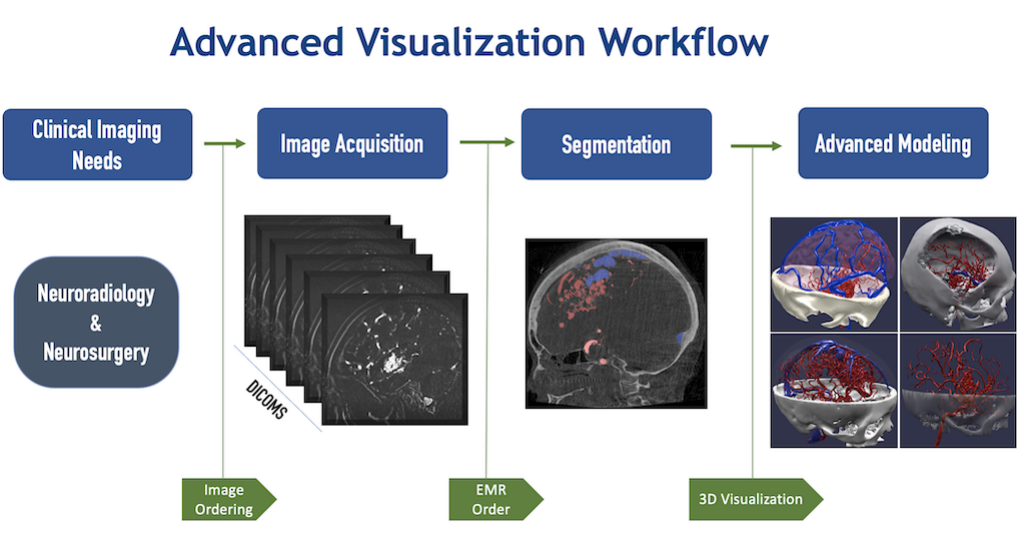
Ochsner BioDesign Digital Request and Fabrication Process. An electronic medical record–based order request is placed by a clinician. Source imaging DICOM (Digital Imaging and Communications in Medicine) datasets are accessed via a secure enterprise PACS (Picture Archiving and Communications System). Biomedical engineers utilize computer-aided design applications to transform DICOM datasets into 3D file types. These 3D files are then either visualized using a web-based browser or extended reality modalities such as virtual or augmented reality. These 3D files can be further processed to produce physical models using 3D printing. DICOM: Digital Imaging and Communications in Medicine; EMR: electronic medical record.

Clinicians request AV modeling based on routine clinical neuroimaging imaging, for example, computed tomography, magnetic resonance, or rotational angiography via an electronic medical record order set created to request patient-specific 3D AV models. Anatomical models are based on the digital imaging and communications in medicine (DICOM) datasets used in clinical imaging. DICOM data are accessed by a staff biomedical engineer via a compliant and secure enterprise picture archiving and communications system. The biomedical engineer segments these DICOM datasets by using computer-aided design software to produce 3D mesh files, specifically standard tessellated language files. Segmentation is the process by which regions of interest are identified through automatic methods (eg, thresholding, edge detection, region growing) and are then refined by manual selection and separation of anatomical structures. This step is completed by the biomedical engineer in conjunction with a radiologist, neurologist, or neurosurgeon to ensure an accurate representation of the region of interest. Once created, these 3D models are rendered and manipulated digitally via an internally developed web-based viewer. These digital models can be further postprocessed for 3D printing to create physical models (Figure 1).

### Epidemiological Data Collection

A CVD AV registry is used to collect data on clinical and utilization metrics associated with AV model use via a compliant research data capture application. Data are collected from the electronic medical record and include patient age at the time of modeling, sex, race, insurance status, imaging modality, comorbidities (hypertension, diabetes, epilepsy, tobacco use), size/scale/location of iAVM, associated current procedural terminology (CPT) codes, presence and type of any associated and relevant procedure within 9 months of model request, the presence and duration of any associated intensive care unit stay, as well as a Spetzler-Martin Grade (SMG). SMG is a clinical tool to assess the procedural risk of iAVMs that incorporates the size, location, and type of venous drainage.

### Clinician and Model Survey Data Collection

Twenty-four clinicians were invited to participate in this study from the organization’s neuroscience institute via clinical and educational conferences, 18 of whom responded. Each clinician assessed a set of 10 sequential iAVM cases, which had previously been requested for modeling. Each participant received a secure email, which included a link to the model utility survey as well as the following information to review: (1) a traditional 2D viewer with the axial, sagittal, and coronal source images available in serial via an embedded digital multimedia container format file, specifically an mp4; (2) information to access and view the source imaging via the standard picture archiving and communications system viewing approach; (3) the associated anatomical 3D AV model accessible via an internally developed web-based viewer; and (4) the radiographic report of the imaging from which the model was created.

Drawing from similar instruments used in the literature, a survey is used to assess (1) demographics and level of experience, (2) clinician-reported complexity of each case on 1-10 Likert Scale, and (3) utility of the patient-specific AV models using a 1-10 Likert scale, with higher scores representing increased complexity and utility (Multimedia Appendices 1-3) [10,12-16]. The survey includes questions regarding the potential diagnostic and therapeutic impact of 3D AV models compared to standard 2D representations. The research data capture application was used to digitally distribute and collect survey data in an automated and compliant way.

### Analysis Plan

Descriptive statistics (averages, range, frequency) were reported for the epidemiological and utilization data for the AV models as well as the demographic and experience data of the survey respondents. Model survey responses were treated as ordinal. Each respondent’s complexity and utility response was averaged to mitigate the limitations associated with the statistical analysis of the dependent and repeated measurements. The independent variable is experience level, and 2 approaches were used to classify experience groups. One approach categorizes experience by training level: (1) trainee defined as medical student, resident, or fellow; (2) staff as defined by those who have completed their terminal clinical training; and (3) advanced practice provider (APP). An alternative approach categorizes experience based on the reported number of iAVM evaluations: low (0-10), medium (11-100), and high (>101). The dependent variables are reported complexity and utility of the AV model compared to traditional 2D viewing. The nonparametric Kruskal-Wallis H test was used to assess the null hypothesis that there is no difference in reported complexity or utility of an AV model among clinicians in these 3 groups. A significance level or α of .05 was used for statistical testing. A Dunn multiple comparison test was used for post hoc analysis to compare specific experience category combinations. A significance level or α of .05 was used for statistical testing.

### Ethics Approval

This study was reviewed and approved by the Ochsner institutional review board (approval 2019.089) and was determined to be exempt and granted a waiver of informed consent, as it posed minimal risk to participants. All data collected for this study were fully deidentified prior to analysis to ensure participant privacy and confidentiality. Study models and data were deidentified and anonymized. Secure storage protocols were implemented in compliance with institutional and federal data protection standards. Access to the data was restricted to authorized study personnel directly involved in data collection and analysis. No compensation was provided to study participants. All procedures were conducted in accordance with appropriate ethical guidelines and applicable regulations.

## Results

The aim of this study was to deploy a digital fabrication process that can (1) describe the clinical attributes associated with the use of AV models and (2) assess the utility of AV models across clinical experience levels when compared to standard 2D viewing.

### Epidemiology and Utilization

AV models for 26 patients with iAVMs were requested from July 2017 to February 2023. Of the 26 models requested, 25 AV models were created. One request was not modeled because the primary source image was ordered by the clinician but never completed by the patient. Of note, 6 out of the 25 cases were also 3D printed to create physical patient-specific models (Multimedia Appendix 4). Of the AV models, 60% (15/25) were based on rotational angiography, 32% (8/25) on computed tomography angiography, and 8% (2/25) on magnetic resonance angiography. Patient age ranged from 13 to 71 years (mean age 37.4 years, SD 16.4 years) with various comorbidities and diverse demographics (Table 1).

**Table 1:** Demographics and comorbidities of the participants for whom 3D models were created.

				Values, n (%)
**Smoking (n=25)**				
	Nonsmoker			4 (16)
	Smoker			21 (84)
**Comorbidities (n=10)**				
	Hypertension			8 (80)
	Diabetes mellitus			2 (20)
	Hyperlipidemia			0 (0)
	Seizure/epilepsy			0 (0)
	Other			0 (0)
**Total comorbidities (n=21)**				
	1			8 (38)
	2			4 (19)
	3			6 (29)
	4			1 (5)
	5			2 (10)
**Other epidemiology metrics (n=25)**				
	**Gender**			
		Female	8 (32)	
		Male	17 (68)	
	**Race**			
		Caucasian	14 (56)	
		African American	11 (44)	

**Figure 2:**
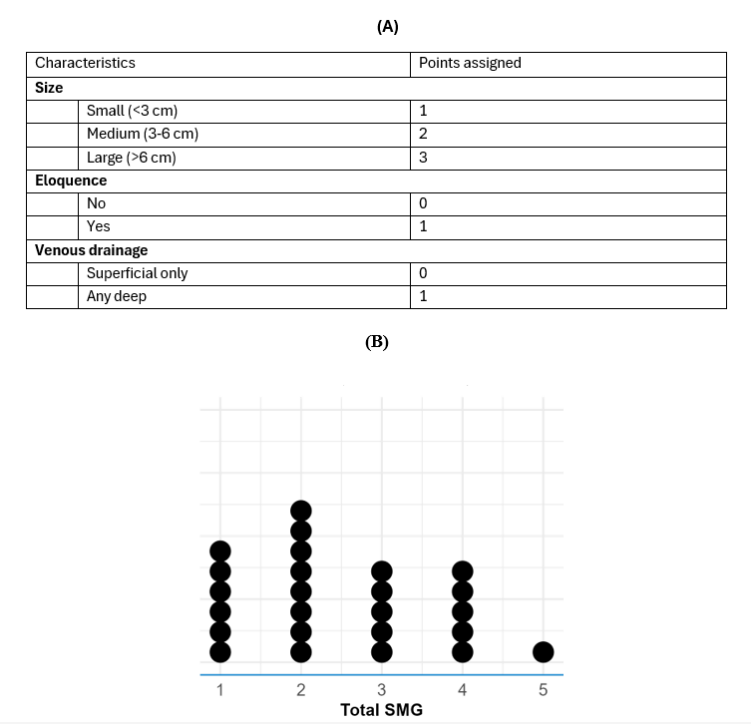
(A) Spetzler-Martin Grading scoring approach and (B) dot plot of Spetzler-Martin Grading scores, showing the distributions for values 1 (6/25, 24%), 2 (8/25, 32%), 3 (5/25, 20%), 4 (5/25, 20%), and 5 (1/25, 4%). SMG: Spetzler-Martin Grading.

With regards to pathophysiology, iAVM diameter ranged from 4.5 mm to 62 mm, with an average diameter of 29.2 mm. Intracerebral hemorrhage was associated with 28% (7/25) of the cases. SMG scores were obtained and ranged from 1 to 5 with a median score of 2 (Figure 2).

The most frequent iAVM location was the frontal lobe (9/25, 36%), and the most frequent venous draining was the superior sagittal sinus (9/25, 36%) (Table 2). Clinically, 56% (14/25) of the models were associated with some form of open or endovascular intervention within 9 months of the AV model request.

With regard to utilization, 9 unique CPT codes were found to be associated with these models. The most frequent CPT code associated with AV models was for selective unilateral catheter access of the common carotid artery (36223) and was billed in 84% (21/25) of the cases. Of the patients who had models created, 64% (16/25) had public insurance, with most patients utilizing Medicaid (14/25, 56%). Intensive care unit stays were associated with 56% (14/25) of the cases with a mean length of 6.9 days and a maximum length of 34 days. The 25 AV models were created for 4 unique clinicians at the following frequencies: interventional neuroradiologist (1/25, 4%), pediatric neurosurgeon (2/25, 8%), interventional neurologist (5/25, 20%), and a vascular neurosurgeon (17/25, 68%).

**Table 2:** Frequency distribution of intracerebral arteriovenous malformation locations and venous drainage categories (n=25).

Location/category			Values, n (%)
**Intracerebral arteriovenous malformation locations**			
	Frontal lobe	9 (36)	
	Frontoparietal lobe	5 (20)	
	Occipital lobe	3 (12)	
	Other	8 (32)	
**Venous drainage category**			
	Superior sagittal sinus	9 (36)	
	Cortical vein	5 (20)	
	Transverse sinus	3 (12)	
	Sigmoid sinus	2 (8)	
	Other	6 (24)	

### Descriptive Statistics for Clinicians

Of the 24 clinicians who completed the initial recruitment survey between November 2022 to January 2023, 18 completed at least one model assessment survey. Six clinicians were lost to follow-up, that is, 1 medical student and 5 APPs. The descriptive statistics of the demographics and experience were calculated for the 18 clinicians who responded (Table 3). Clinicians (mean age 36.5 years; median age 34.5 years, range 26-56 years) identified their specialties as neurosurgery (1/18, 6%), neurointerventional radiology (3/18, 17%), and neurology (12/18, 67%). Two respondents (11%) were medical students and did not identify with a specialty. The other respondents identified as residents (4/18, 22%), APPs (5/18, 28%), fellow (1/18, 6%), junior attendings with <5 years of experience from terminal training (3/18, 17%), and senior attendings with >5 years of experience after terminal training (3/18, 17%). Self-reported iAVM evaluations was reported as 0 (2/18, 11%), 1-10 (4/18, 22%), 11-50 (6/18, 33%), 51-100 (3/18, 17%), 101-200 (2/18, 11%), and >201 (1/18, 6%).

**Table 3:** Summary statistics of the specialties and level of training for clinician survey respondents (n=18).

		Values, n (%)
**Specialties/level of training**		
	Neurosurgery	1 (6)
	Neurointerventional radiology	3 (17)
	Neurology	12 (67)
	Medical student	2 (11)
**Level of training**		
	Trainees	5 (28)
	Staff	6 (34)
	Advanced practice provider	5 (28)
**Number of arteriovenous malformation evaluations**		
	Low (<10)	6 (34)
	Medium (51-100)	9 (50)
	High (>101)	3 (17)
**Clinician gender distribution**		
	Male	10 (56)
	Female	8 (44)

### Descriptive Statistics of Model Surveys

**Figure 3:**
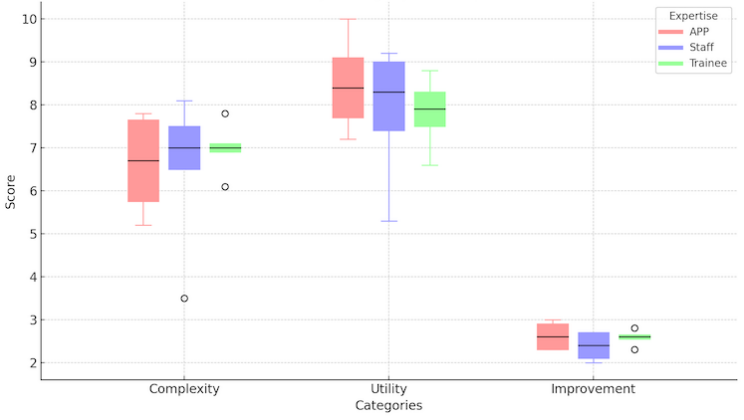
Boxplots for complexity, utility, and improvement derived from 3D model based on expertise: advanced practice provider (n=5), staff (n=6), and trainee (n=7). APP: advanced practice provider.

**Figure 4:**
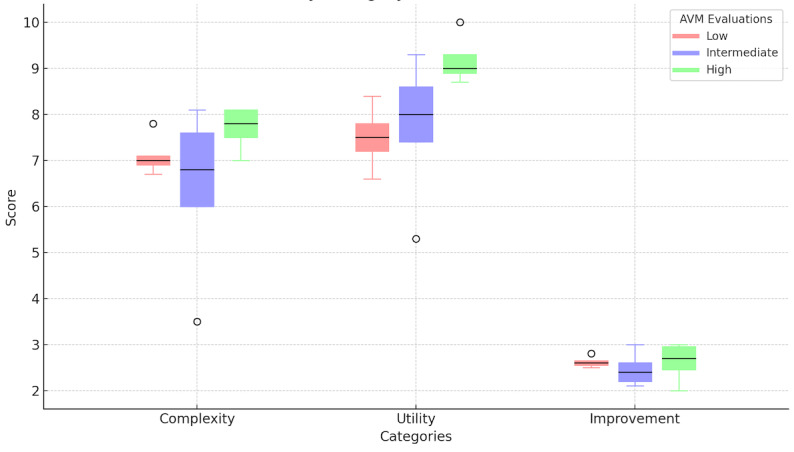
Boxplots for complexity, utility, and improvement derived from 3D model based on the number of arteriovenous malformations evaluated: low (0-10, n=6), intermediate (11-100, n=9), and high (>101, n=3). AVM: arteriovenous malformation.

Each of the 18 clinicians were provided 10 model surveys for a total of 180 possible responses. Approximately 95.6% (172/180) of the surveys were initiated, and 92.2% (166/180) were completed. The possible complexity scores ranged from 1 to 10, with 10 being the most complex. The median of the average complexity reported was 6.95 with a minimum average score of 3.5 and a maximum average score of 8.1 across all respondents. Descriptive statistics, including median and IQR, were calculated for utility, complexity, and improvement attributed to access to AV models for each expertise level (Figure 3) based on training level and the number of iAVMs evaluated (Figure 4). Clinicians responded affirmatively in 99.4% (171/172) of the cases that they would like to share AV models with their patients.

There was a total of 6 physicians who had completed their terminal training with a cumulative survey response rate of 85% (51/60). When compared to standard 2D models, these 6 staff clinicians reported that AV models provided some improvement in visualization in 49% (25/51) of the cases and substantial improvement in 41% (21/51) of the cases. Staff level physicians reported that in 49% (25/51) of the cases, they were able to appreciate normal anatomy in the AV model that they could not in standard 2D imaging. Similarly, staff reported that in 45% (23/51) of the cases, they were able to appreciate abnormal anatomy in the AV model that they could not in standard 2D imaging. Staff answered affirmatively in 14% (7/51) of the cases that the AV model would change their diagnosis while reporting affirmatively in 45% (23/51) of the cases that the AV model would change their therapeutic approach (Table 4).

**Table 4:** Diagnostic and management impact of advanced visualization compared to traditional 2D viewing on staff level clinicians (n=51).

		Values, n (%)
**Change in diagnosis**		
	No	39 (76)
	Yes	7 (14)
	No response	5 (10)
**Change in therapeutic procedures**		
	Yes	23 (45)
	No	21 (41)
	No response	7 (14)
**Change in appreciation of normal anatomy in 3D**		
	Yes	25 (49)
	No	20 (39)
	No response	6 (12)
**Change in appreciation of pathologic defects in 3D**		
	Yes	23 (45)
	No	22 (43)
	No response	6 (12)

### Inferential Analytics of Model Surveys

**Figure 5:**
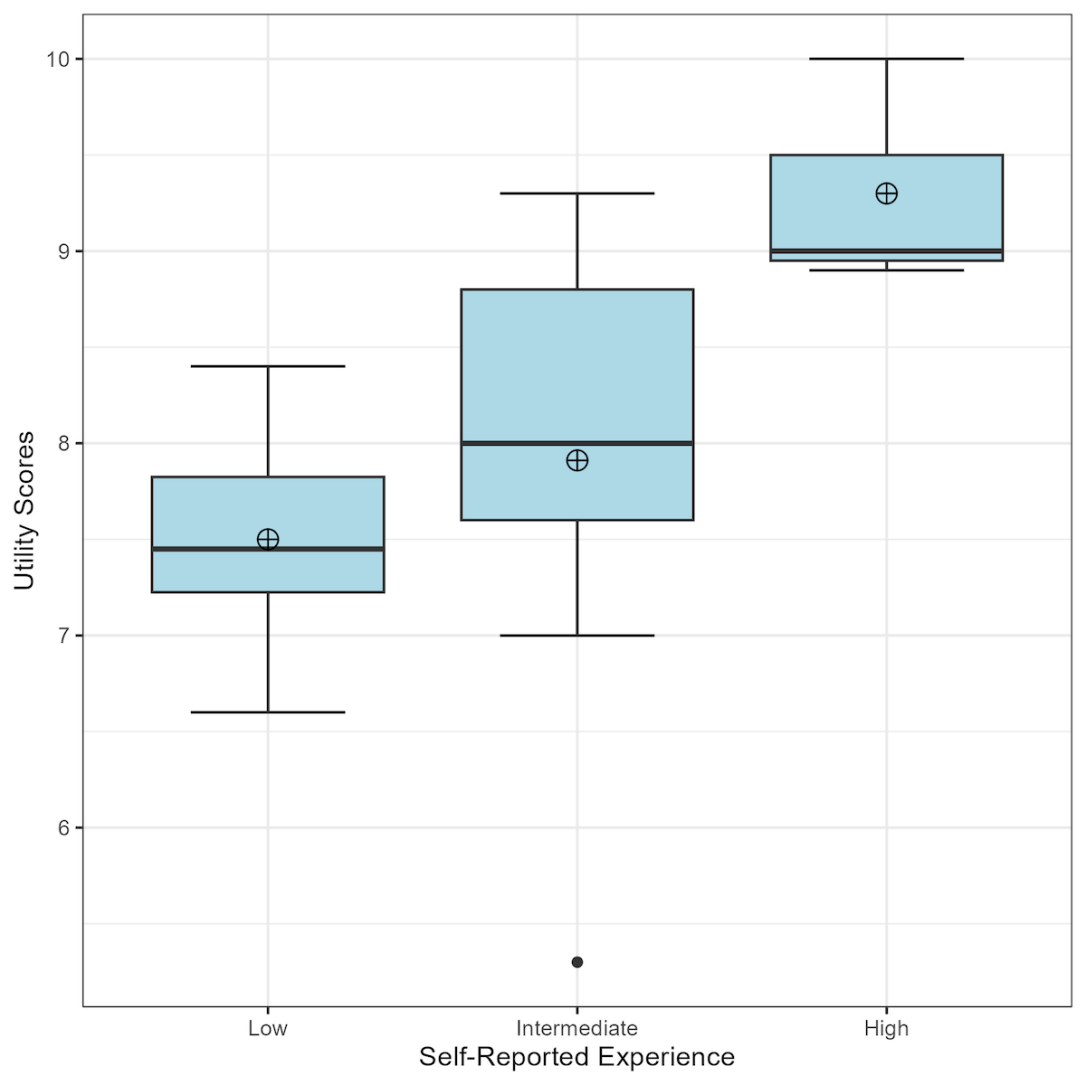
Boxplot of utility across self-reported experience. The Kruskal Wallis H test demonstrated a statistical difference between the low and high experience groups (*P*=.03). The analysis reveals that the median utility scores for low (n=6), intermediate (n=9), and high (n=3) categories of self-reported experience are 7.45, 8.00, and 9.00, respectively.

When categorized by training level, the 3 groups were distributed between APPs (5/18, 28%), trainees (7/18, 39%), and staff (6/18, 33%). The Kruskal-Wallis H nonparametric test was deployed with nonsignificant *P* values >.05 for the survey questions relevant to model complexity (*P*=.72) and utility (*P*=.48). Thus, we fail to reject the null hypothesis. Rather, we conclude that there is no statistically significant difference in reported complexity nor utility between clinician types. When categorized by self-reported experience, the 3 groups were distributed between low (6/18, 33%), intermediate (9/18, 50%), and high (3/18, 17%). The Kruskal-Wallis H nonparametric test was deployed with *P*=.12 for the survey questions relevant to model complexity. Thus, we fail to reject the null hypothesis for complexity and conclude that there is no statistically significant difference in reported complexity between experience levels. The *P* value for utility, however, did achieve significance with *P*=.03. Thus, for utility, we can reject the null hypothesis and conclude that there is a statistically significant difference in the reported utility metric between self-reported experience groups. Post hoc analysis with Dunn multiple comparison test demonstrated that the comparison between the low and high group showed a significant difference in the utility metric. There was not a significant difference in the utility metric between the comparisons of the other groupings: high-intermediate or intermediate-low (Figure 5).

## Discussion

### Principal Findings

This study demonstrates the feasibility of creating and deploying a digital fabrication and assessment infrastructure for AV models in complex presentations of CVDs, such as iAVMs. The scalable and secure request, creation, distribution, and evaluation of AV models was confirmed by the implementation of 25 AV models for iAVMs from July 2017 to February 2023 at a single high-volume certified stroke center. This retrospective analysis confirmed the utilization of AV models in CVDs, specifically iAVMs. Multiple clinicians requested AV models that were created from a variety of imaging modalities, most commonly from rotational angiography. Of note, requests for AV models persisted despite the decrease in elective procedures during the COVID-19 pandemic and a nationwide iodinated contrast shortage in 2022, which severely impacted imaging and procedure volumes and is indicative of the utility clinicians derive from AV models.

This analysis demonstrates associations between AV model use in CVD and a diverse set of clinical attributes and utilization metrics. Consistent with the complex and diverse population in Louisiana and the surrounding Gulf Coast region, a substantial number of models were created for individuals younger than 40 years, African Americans, and patients on Medicaid. Many of these patients were not only young but otherwise healthy with few if any other comorbidities, besides tobacco use. The feasibility to collect health care finance and utilization metrics such as CPT codes and intensive care unit lengths of stay associated with the request for AV models in complex CVD presentations was confirmed. The analysis revealed the association of AV models with resource-intensive health resources such as intensive care unit stays, rotational angiography, and neurovascular procedures.

A survey instrument grounded in the literature was successfully created and implemented to assess the impact of AV models in complex CVD at scale. There was strong engagement across a variety of clinical experience levels, particularly APPs, neurological residents, and neurovascular staff, with an overall 92.2% (166/180) survey completion rate. On a set of iAVMs of varying complexity, a diverse array of clinicians reported substantial utility of AV. Staff-level clinicians reported that AV models would have rarely changed their diagnosis compared to traditional 2D viewing. Staff, however, reported that in nearly half of cases, the patient-specific AV models allowed them to appreciate normal and abnormal anatomy that they could not with traditional 2D imaging. Moreover, in 45% (23/51) of the cases, access to the AV model would have changed their therapeutic approach compared to their assessment based on traditional 2D viewing. Staff physicians reported that AV models were much more likely to change their therapeutic approach than their diagnosis. There was no significant difference in reported complexity or utility when clinicians were categorized by training: APP, trainee, staff. There was, however, a statistically significant difference (*P*=.03) in reported utility when clinicians were categorized by self-reported experience. Interestingly, the difference in reported utility was most pronounced between the high and low experience group, with the most experienced group reporting more utility than those with less experience. This was contrary to our hypothesis and suggests that the ability to understand the idiosyncratic and complex pathologies and extract relevant information from AV models may require a certain level of pre-existing knowledge and clinical expertise. Such a prerequisite appreciation of clinical anatomy may help clinicians derive diagnostic and therapeutic insights from AV models. Alternatively, this observation may be attributable to the psychological phenomena known as the Dunning-Kruger effect in which people with low ability, expertise, or experience regarding a certain type of task or area of knowledge tend to overestimate their ability or knowledge [17].

### Strengths and Limitations

This study has multiple strengths attributable to a multidisciplinary approach that focused on clinical operational integration as a foundation for clinical research. Evaluation of an AV intervention required coordinating a cross-functional team consisting of biomedical engineers, research staff, clinical administrators, and clinicians. This study was the impetus to expand and ruggedize a scalable digital fabrication and assessment process for AV models. The survey response rates indicate not only enthusiasm for the field of inquiry but the willingness of these busy and increasingly overextended clinicians to engage in clinical research on 3D AV [18,19]. This points to the importance of continuing to develop and validate AV technologies not just for CVDs but for other complex disease states, including but not limited to spine deformity, genitourinary pathologies, orthopedic trauma, craniomaxillofacial abnormalities, and congenital heart disease.

This study has several limitations. This was a nonrandomized study, which could only establish associations and not causative impacts of AV modeling. The ability to extrapolate these findings is also limited by its small sample size and focus on a single center. Clinician respondents were basing feedback on hypothetical use cases and not the actual implementation of the 10 models in diagnosis or treatment planning. Moreover, the clinicians were all colleagues who may have influenced each other’s responses, which may have introduced a diffusion of treatment effect as described by Urban and van Eeden-Moorefield [20]. Finally, the results of the statistical testing may be due to the small sample size rather than the absence or presence of differential utility among varying experience levels.

### Challenges and Future Directions

The creation of AV models remains resource-intensive in terms of expertise, time, and infrastructure. Complex disease states where AV models are likely to be the most impactful are often low in prevalence requiring longer periods of study and collaboration between multiple sites, engendering a gap in the literature. These barriers have limited the number of conclusive studies validating the clinical and utilization impact of 3D AV models. Approaches to increase the efficiency of AV model creation, for example, machine-learning enhanced segmentation, may mitigate these obstacles to validation and warrant further research and development.

This study expands and utilizes a scalable digital fabrication infrastructure for future larger multisite randomized studies to assess the efficacy of AV modeling. Such future trials could examine the utility of AV models in clinical care delivery by incorporating technical resource requirements such as segmentation time, utilization measures such as operative time and radiation exposure, as well as clinical outcome metrics such as functional status and hospital stay. This study establishes the feasibility of collecting medical billing and utilization metrics associated with 3D AV models that can be used in future cost-effective analyses. This infrastructure could also be applied to understand the use and impact of AV models in other disease states that would benefit from AV techniques. Future studies could incorporate quantitative and qualitative assessments of other AV modalities such as 3D printing or extended reality to investigate the different applications and utility of digital versus physical models in visualization and simulation. Finally, this infrastructure could be utilized to study the impact AV models have on health literacy and patient experience. The use of AV is quickly being adopted in clinical training and care delivery but requires further investigation and validation.

### Conclusion

This pilot feasibility study establishes the infrastructure to create and assess patient-specific AV models in a digital and scalable way. This study confirms the use of AV models in complex, resource-intensive disease states such as iAVMs. This study demonstrates enthusiasm for the use of AV across different specialties and experience levels involved in the management of CVD. A heterogenous cohort of clinicians reported substantial utility of AV modeling when compared to standard 2D imaging. Counter to our hypothesis, those clinicians with the most experience found AV models the most useful. Finally, staff level physicians reported at high rates that AV models modified therapeutic approaches and provided anatomical insights not appreciated in standard 2D imaging. Almost universally, clinicians across different specialties and experience levels affirmed that that their patients would benefit from the ability to view their pathology-specific 3D AV models. Collectively, these findings support the idea that compared to traditional 2D viewing, patient-specific AV models are useful to a diverse array of clinicians involved in the treatment of complex disease states, such as iAVMs.

## Appendix

Multimedia Appendix 1 Survey template: clinician, on-screen model survey, and survey email template.

Multimedia Appendix 2 Representative 2D viewer included in surveys.

Multimedia Appendix 3 Representative web-based 3D viewer included in surveys.

Multimedia Appendix 4 Photograph of 3D printing of an intracerebral arteriovenous malformation example.

## Abbreviations

APP: advanced practice provider
AV: advanced visualization
CPT: current procedural terminology
CVD: cerebrovascular disease
DICOM: Digital Imaging and Communications in Medicine
iAVM: intracerebral arteriovenous malformation
SMG: Spetzler-Martin Grade

## Data Availability

The datasets generated during and analyzed during this study are available from the corresponding author on reasonable request.
