# Supplementary material for: Assessing the Feasibility and Utility of Patient-Specific 3D Advanced Visualization Modeling in Cerebrovascular Disease: Retrospective Analysis and Prospective Survey Pilot Study
Source: JMIR Form Res. 2025 Feb 21;9:e51939. doi: 10.2196/51939 (PMC11890146; doi:10.2196/51939)
Supplement: Multimedia Appendix 1 [file formative_v9i1e51939_app1.docx]

**Appendix 1 Survey Template: Clinician Subject, On-Screen Model Survey, & survey e-mail template**

Clinician Subject E-mail

Thank you for agreeing to participate in our study to assess the impact of 3D Models in Cerebrovascular Disease. Please complete this preliminary survey to help us collect demographics and experience level data for our study. I appreciate you taking the time and look forward to working with you.

Clinician Subject Survey

1. What is your age?
2. What is your Gender? Male, Female, Other
3. What is your training level: Medical Student, Resident, Advanced Practice Provider, Fellow, Junior Attending (<5 years from terminal training), Senior Attending (> 5 years from terminal training)
4. What is your Specialty: Neurosurgery, Neurointerventional Radiology, Neurology, Neurocritical Care, Other
5. Approximately how many aneurysms evaluations have you been involved in during your career, including training: none, 1-20, 21-100, 101-200, 201-500, >500
6. Approximately how many Arteriovenous Malformations evaluations have you been involved in during your career, including training: none, 1-10, 11-50, 51-100, 101-20, >201

Model Review Survey

Thank you for participating in the Cerebrovascular Disease Advanced Visualization study.

1. Please Review the original 2D imaging with the attached video. If you would like to review the original 2D imaging ***via Epic*** further you can use the following information: *Medical Record Number* (*MRN), accession number, and date of acquisition*
2. Review the attached on-screen 3D Viewer
3. Review the attached radiographic report

Please then complete this *linked survey*

Model Survey

1. How complex would you rate this case? NOT complex 1  2 3 4 5 6 7 8 9 10    VERY Complex

2. How helpful did you find the on-screen 3D Model? NOT complex 1  2 3 4 5 6 7 8 9 10    VERY Complex

3a. Were you able to appreciate any normal anatomy in 3D that you were not able to in 2D? Yes/No

b. Did you appreciate any abnormal pathological defects in the 3D model that you were not able to in 2D? Yes/No

4. Does 3D modeling, either with physical models or with on-screen models, improve your ability to visualize the anatomy?

- No additional improvement
- Some additional improvement
- Substantial additional improvement

5. Did the 3D model change your

a. Diagnosis: Yes   No

b. Therapeutic and/or procedural approach, Yes   No

6.Please ***estimate*** how much time is saved in the OR by having access to a patient-specific 3D Model:

- none
- 0-30 min
- 30-60 min
- 60-120 min
- >120 min

7. Do you believe your patients would like to view their 3D model? yes/no
