# Supplementary material for: Assessing the Feasibility and Utility of Patient-Specific 3D Advanced Visualization Modeling in Cerebrovascular Disease: Retrospective Analysis and Prospective Survey Pilot Study
Source: JMIR Form Res. 2025 Feb 21;9:e51939. doi: 10.2196/51939 (PMC11890146; doi:10.2196/51939)
Supplement: Multimedia Appendix 3 [file formative_v9i1e51939_app3.zip › 317_3D Viewer.html]

m3D 2D Viewer
