# Supplementary figures and images for: Assessing the Feasibility and Utility of Patient-Specific 3D Advanced Visualization Modeling in Cerebrovascular Disease: Retrospective Analysis and Prospective Survey Pilot Study
Source: JMIR Form Res. 2025 Feb 21;9:e51939. doi: 10.2196/51939 (PMC11890146; doi:10.2196/51939)

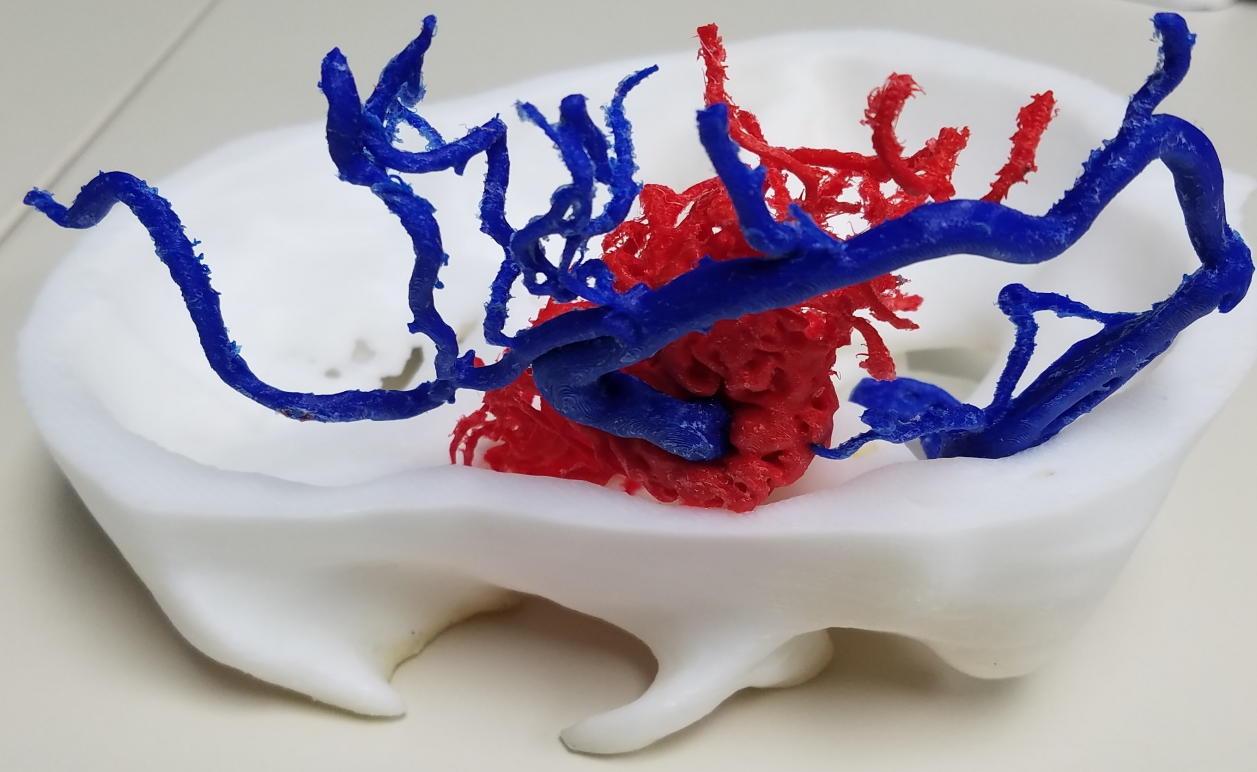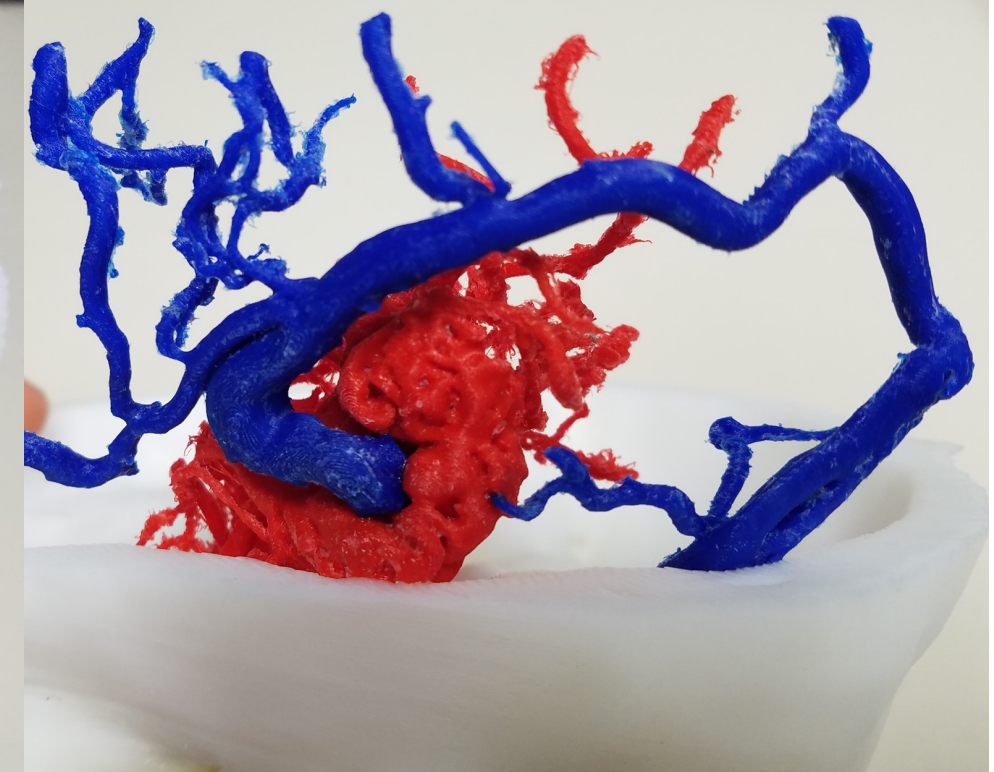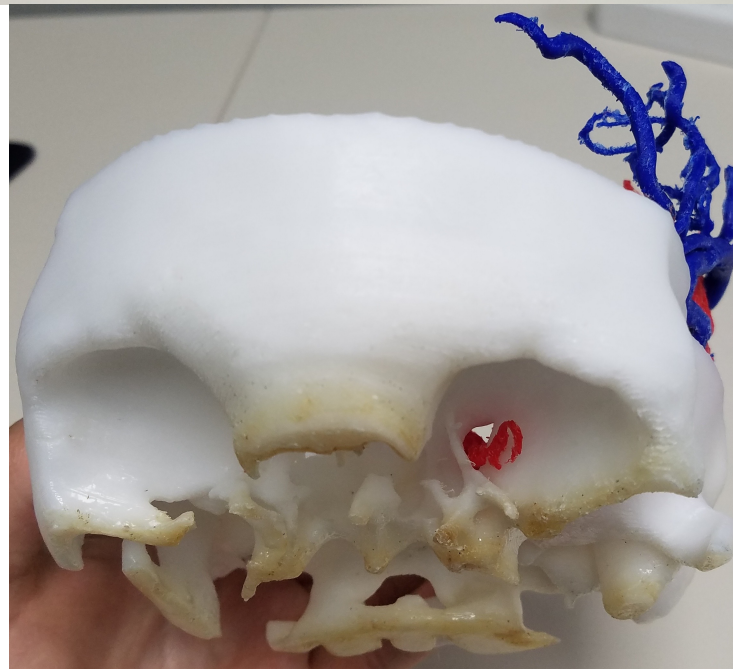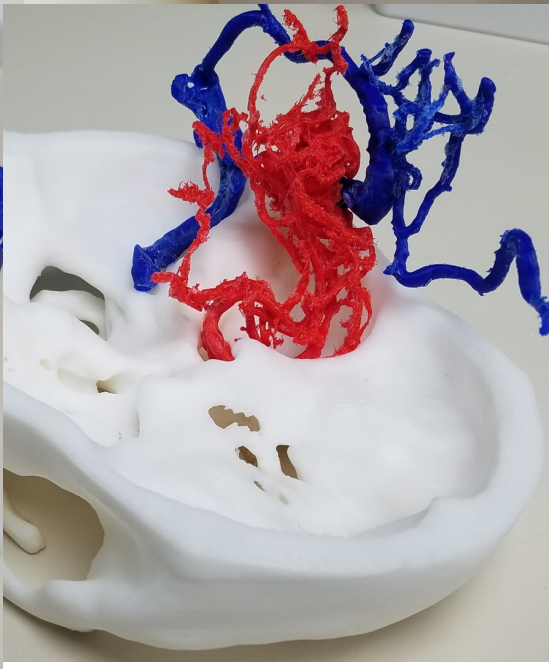

Supplement: Multimedia Appendix 4 [file formative_v9i1e51939_app4.pdf]
